# Micro Plastic Deformation Behavior of Carbide-Free Bainitic Steel during Tensile Deformation Process

**DOI:** 10.3390/ma13214703

**Published:** 2020-10-22

**Authors:** Ming Zhang, Xiaowu Yang, Jie Kang, Fucheng Zhang, Zhinan Yang

**Affiliations:** 1 State Key Laboratory of Metastable Materials Science and Technology, Yanshan University, 438 Hebei Street West Section, Haigang District, Qinhuangdao 066004, China; zhm@ysu.edu.cn (M.Z.); yxw1161107129@163.com (X.Y.); fczhang08@163.com (F.Z.); 2 School of Materials Science and Engineering, Hebei University of Science and Technology, 26 Yuxiang Street, Yuhua District, Shijiazhuang 050018, China; Jiekang@hebust.edu.cn; 3 National Engineering Research Center for Equipment and Technology of Cold Strip Rolling, Yanshan University, 438 Hebei Street West Section, Haigang District, Qinhuangdao 066004, China

**Keywords:** bainitic steel, in-situ analysis, plastic deformation, strain-induced transformation, strain distribution

## Abstract

The microstructure of carbide-free bainitic steel has been widely reported because of its excellent mechanical properties. However, no work has explored the deformation behavior of each phase in the microstructure, namely, retained austenite (RA) and bainitic ferrite (BF). In this paper, the deformation behavior of RA and BF during tension was investigated through the in situ electron backscatter diffusion method. The results showed that the RA surrounded by BF with a high Schmid factor (SF) was accelerated towards being transformed into martensite, but the surrounding BF with low SF value retarded the transformation. The kernel average misorientation (KAM) findings revealed that the strain of the RA and BF rapidly increased initially, slowed down after the critical points of 0.25 and 0.54, respectively, and then increased again. The calculated result for the KAM fraction indicated that the contribution of RA to the total plastic deformation gradually decreased throughout the total tensile process, whereas that of BF increased in the presence of RA.

## 1. Introduction

Carbide-free bainitic steel is mainly composed of bainitic ferrite (BF) and carbon-rich retained austenite (RA) [1,2]. RA is distributed between BF lathes and/or sheaves, possessing different stabilities, and the two phases coordinate with each other during deformation, thereby guaranteeing an excellent balance of strength and toughness, especially when the coarse, blocky RA is eliminated. In bainitic steels, RA is a metastable phase that can transform into martensite because of stress concentration [3,4,5,6]. The stability of RA is influenced by several factors, such as morphology, grain size, carbon content, distribution, and crystalline orientation [7,8]. Knijf et al. modelled the martensite transformation process of 11 kinds of RA with different orientations during uniaxial tensile testing [8], and revealed that transformation potential varies linearly with transformation rate, yielding a correlation coefficient of approximately 0.75. However, the effect of the crystal orientation of the surrounding phase on the transformation behavior of RA was not considered. The evolution of the soft RA in bainitic steel is the main focus of researchers, with minimal concern for harder phases, such as BF or martensite [3,7,8]. In dual-phase steel, deformation is mainly borne by the soft phase. For carbide-free bainitic steel, RA is the soft phase, but this phase only comprises a small portion of the microstructure. Therefore, deformation should mainly occur in the harder phase BF. However, the distribution of strain and stress between BF and RA in bainitic microstructure during deformation has not been studied in detail.

The strain distribution between RA and BF is heterogeneous because of their dissimilar mechanical properties. Numerous methods have been applied in studying the strain distribution behavior of dual phase (DP) steels. One widely used method in this field is simulation, such as the C-J method, which was formulated by Nie et al. [9]. Wei et al. established a physical model of martensitic-BF steel, and revealed that the grain size and phase volume fraction significantly influenced strain distribution [10]. Varshney et al. proposed that the strain hardening capability of phases also influences the strain distribution behavior [11]. The gradually improving in-situ technique can directly provide results on the strain distribution between each phase [12].

The plastic deformation of RA and BF is mainly achieved by the generation and slipping of dislocations. Therefore, the evolution of dislocations during deformation is crucial in plastic deformation research. Dislocations can be divided into statistically stored dislocations (SSDs), and geometrically necessary dislocations (GNDs) [13,14]. SSDs consist of dipoles and multipoles, and result in the absence of curvature of the crystal lattices. GNDs represent excess dislocations stored within a Burger’s circuit, and contribute to lattice curvature; these dislocations coordinate the total deformation of the material, and result in a gradient distribution of strain in the material [14]. Moreover, GNDs cause material hardening by increasing the total dislocation density [15]. Therefore, research on GNDs facilitates the study of plastic deformation behavior. The electron backscatter diffraction (EBSD) technique has been widely used for GND measurement. Field et al. found that the orientation gradient in deformed grain can be presented by the GND density [16]. Jiang et al. found that the strain of crystalline material exhibits a relative stable function with the logarithm of the GND density (log-GNDs); their findings indicated that the log-GNDs increase with increasing strain and finally stabilize [17]. In this paper, in-situ EBSD was introduced to analyze the strain hardening, strain distribution behavior, and strain-induced martensitic transformation behavior of a carbide-free bainitic steel at the microscale, to ultimately reveal the microplastic deformation behavior of each phase.

## 2. Experimental Methods

The chemical composition of the carbide-free bainitic steel is listed in Table 1.

**Figure 1:**
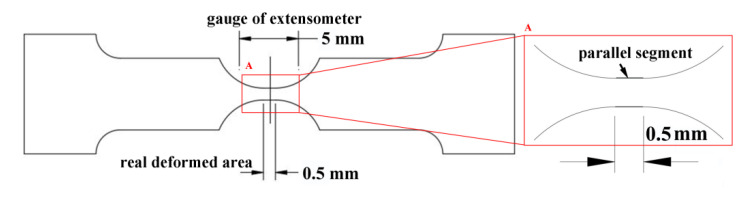
Schematic of the tensile specimen.

The studied steel was smelted in a 45 kg vacuum induction furnace and forged into a round bar (Φ 65 mm). The martensite start transformation temperature (M_s_) of the specimen was 315 °C. Specimens were austenitized at 930 °C for 45 min, subjected to isothermal transformation at 350 °C for 2 h to obtain the carbide-free bainitic microstructure, and then air cooled to room temperature. Finally, all specimens were tempered at 320 °C for 1 h. The specimens were cut into plates with a thickness of 2 mm for tensile testing; the specimen size is shown in Figure 1. To analyze the same area under different strains, we limited the examined deformed area to a parallel section of 0.5 mm length in the middle of the specimens. The tensile strain of the specimen was determined by Equation (1):

(1) $$\varepsilon =\frac{g}{l}d$$

where *ε* is the strain of the specimen, *g* is the gauge of the extensometer, *l* is the undeformed length of the analyzed area, and *d* is the displacement of the extensometer.

**Figure 2:**
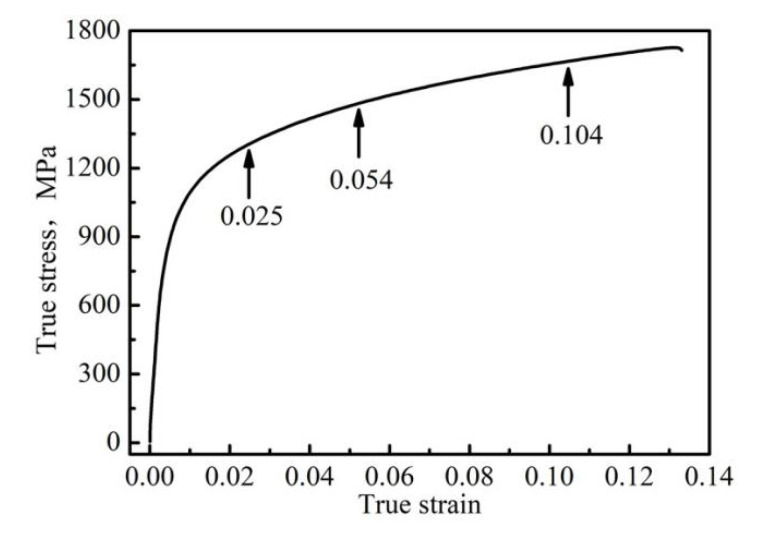
True stress–strain curve of the specimen.

The tensile rate is 2 mm·min^−1^. The accumulative strain of each step was 0.025, 0.054, and 0.104, respectively. Figure 2 shows the continuous tensile curve of the specimen.

**Figure 3:**
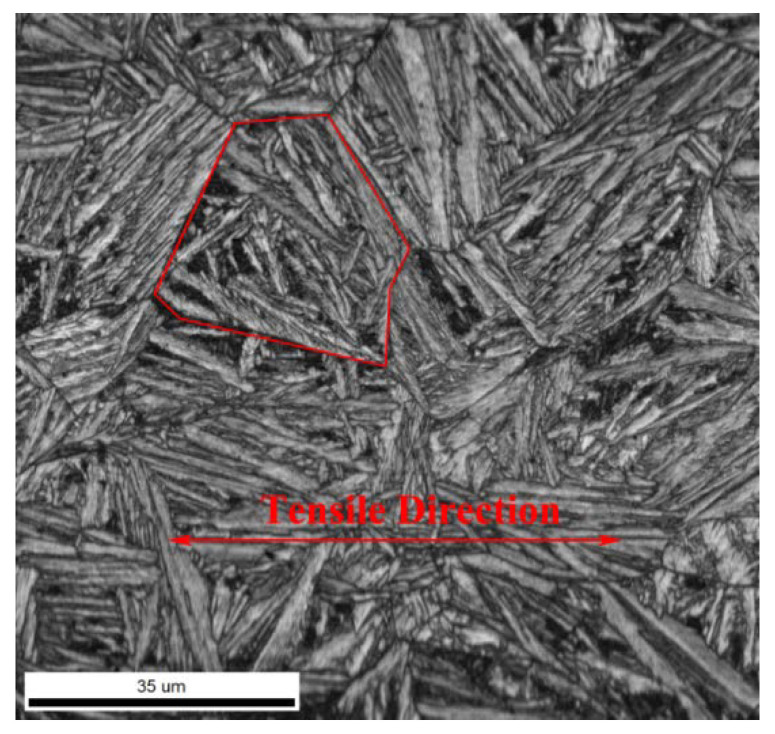
The image quality map of the specimen. Note: the grain encircled by the red polygon is the analyzed region.

An ion figuring system was used to produce a high-quality surface of the specimen for EBSD observation. The mechanically polished sample was jetted by an ion beam for 15 min. The real deformed area was analyzed using EBSD, attached to a scanning electron microscope. EBSD data were acquired on a hexagonal scan grid under an accelerating voltage of 30 kV, tilt angle of 70°, and step size of 80 nm. Raw orientation data obtained at each loading step were post-processed using the OIM Analysis 7.3 software. Orientation, strain, and stress were diverse at different grains and complicated the research. Therefore, the analyzed region was limited to one austenite grain, as indicated by the encircled red polygon of Figure 3. The volume fraction of the retained austenite in specimens with different strain was analyzed by a D/max-2500/PC X-ray diffractometer (XRD, Rigaku, Tokyo, Japan) with CuKα radiation.

## 3. Results and Discussion

### 3.1. Strain-Induced Martensitic Transformation of RA

**Figure 4:**
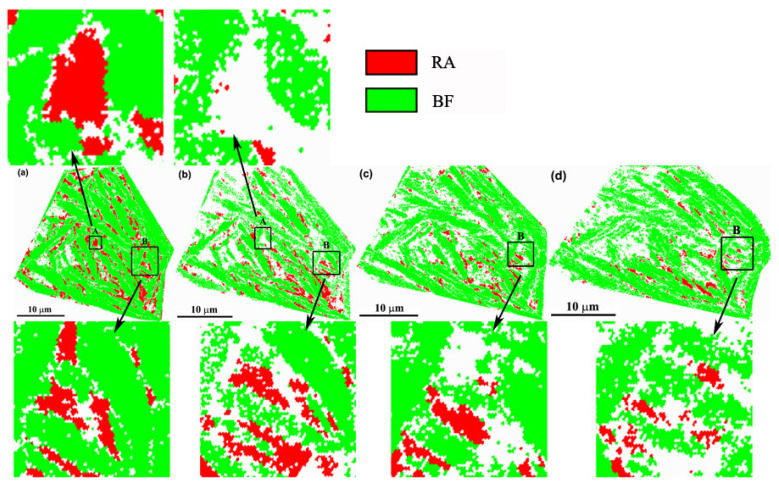
Phase maps of specimens with different strains: (**a**) 0, (**b**) 0.025, (**c**) 0.054, and (**d**) 0.104. The white areas are the areas without index.

**Figure 5:**
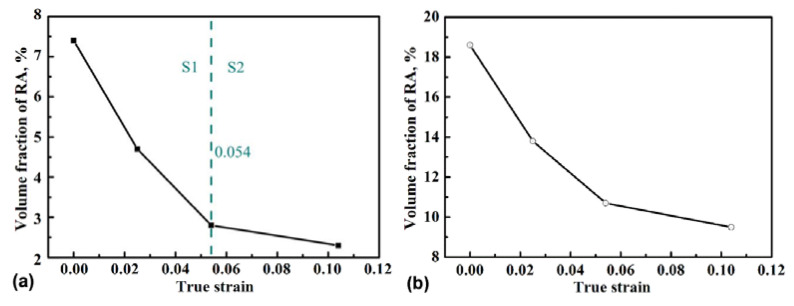
Variation of volume fraction of retained austenite (RA) in the tested region (**a**), and in the specimen tested via XRD (**b**), with true strain.

Figure 4 shows the phase maps of the specimen at different strains. Two types of RA, namely, film-like and block-like, exist within the microstructure. These types possess relatively high and low stabilities, respectively. The amount of RA gradually decreased with increasing strain. Statistical results in Figure 5a reveal two stages for the variation in volume fraction of RA. For stage I, a sharp decrease from 7.5% to 3.0%, with increasing strain from 0 to 0.054, was noted. For stage II, a slow decline from 3.0% to 2.2%, with further increasing strain to 0.104, was observed. The lost RA was transformed into martensite through the strain/stress-induced transformation process [18,19]. Therefore, the mechanical stability of RA is the factor that chiefly affects the variation tendency shown in Figure 5a. In general, the mechanical stability of RA is influenced by carbon content, shape, and size, and the condition of the surrounded phase [7]. The variation of mean volume fraction of the retained austenite in the microstructure, with increasing strain, was estimated via XRD test, as shown in Figure 5b; which shows a similar variation tendency to the tested region. The higher value of the XRD result, as compared with the statistical result from the phase map, is mainly due to the resolution limitations of EBSD, resulting in filmy retained austenite with ultrafine thickness which cannot be revealed in the EBSD phase map.

**Figure 6:**
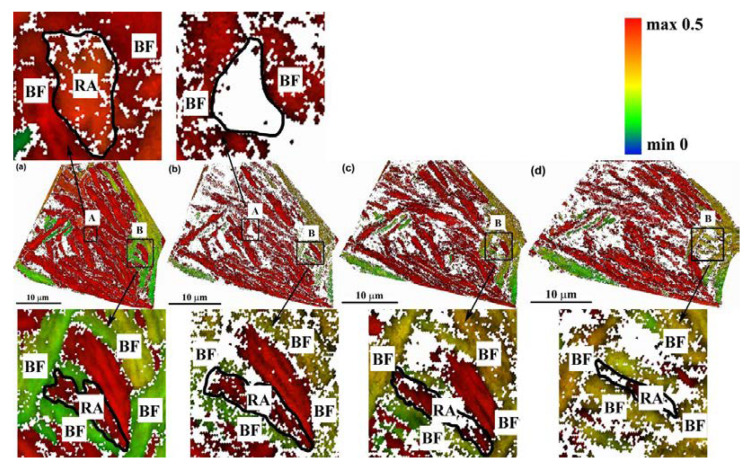
Schmid factor maps in the studied region at different strains: (**a**) 0, (**b**) 0.025, (**c**) 0.054, and (**d**) 0.104.

The Schmid factor (SF) can be used to reflect the potential to activate the slip system in a crystalline. A higher SF value indicates greater ease in activating the slip system than a lower SF value [20]. Figure 6 shows the distribution of SF, where the slip systems of RA were selected as (111)<110>, and those of BF were (110)<111>, (112)<111>, and (123)<111>. The phase in a black irregular closed curve is RA, and the change in SF values are represented by the color gradient.

The SF factor of RA was uniform and high, with a value of about 0.5, which indicated that RA deformation was commenced easily and simultaneously. However, the SF value of BF ranged from 0.2 to 0.5 across different BF plates. In area A, the RA was surrounded by the BF with a relatively high SF, indicating that both RA and BF deformed easily during tension. Stress and strain were easily transferred from BF to RA. Certain studies have also revealed that dislocation within BF can be absorbed by RA [19,21], which not only reserved the soft state of BF, but also further hardened RA. Therefore, the dislocation density within RA easily increased sufficiently to allow martensitic transformation. Then, RA in area A rapidly disappeared when the true strain reached a low value of 0.025 (Figure 6a,b). The condition of RA in area B markedly differed from that in area A. Although RA exhibited a high SF value, that of the surrounding BF was low, at ~0.2. Moreover, the SF value of the surrounding BF in this region maintained a low level during the total tensile process, although a small increase was noted with increasing strain, because of coordination of the neighboring phase. This finding indicated that the BF remained at a hard orientation for dislocation slip, and that increased stress was needed to deform the BF plates. Thus, the stress and strain become difficult to transfer from BF into RA, because RA is shielded by the surrounding hard BF. The RA in area B also became difficult to transform into martensite (Figure 4a–d), and some of the RA was retained when the true strain reached 0.104. Therefore, we conclude that the martensitic transformation of RA was affected by the orientation of the surrounding BF. The low SF value of the surrounding BF retards, whereas the high SF value accelerated, the martensitic transformation.

### 3.2. Microscale Deformation Behavior in RA and BF

**Figure 7:**
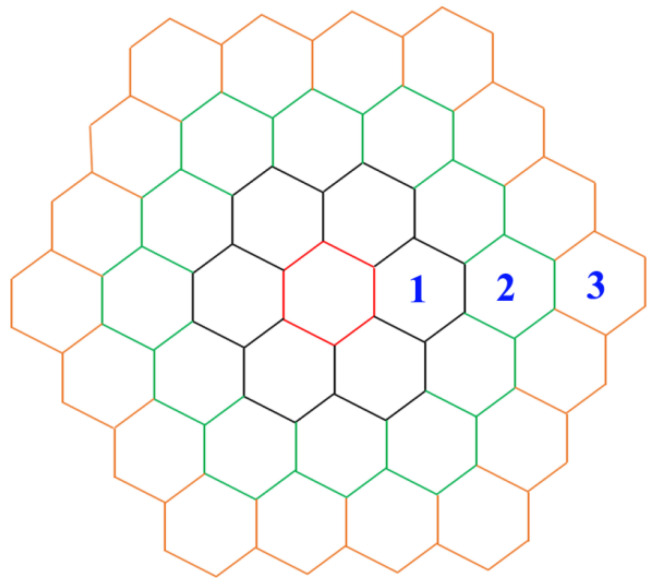
View of the measured points and their neighbors. The red polygon indicates the measured point; the black, green, and yellow polygons represent the first, second, and third adjacent points, respectively.

The deformation behaviors of RA and BF were dissimilar because of their different properties. The strain at the RA and BF can be well characterized by kernel average misorientation (KAM) [22,23,24,25]. A high KAM value represents a large deformation. KAM is the average value of misorientation of measured points and all their neighbors (Figure 7). Normally, the neighbor points within the third adjacent point are included into the calculation process, and the upper tolerance value of misorientation is set as 5° to eliminate the influence of grain boundaries and subgrain boundaries [25,26,27,28,29].

**Figure 8:**
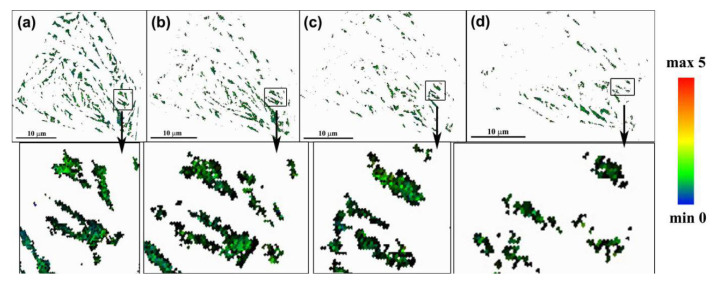
Distribution of kernel average misorientation (KAM) for RA at different strains: (**a**) 0, (**b**) 0.025, (**c**) 0.054, and (**d**) 0.104.

**Figure 9:**
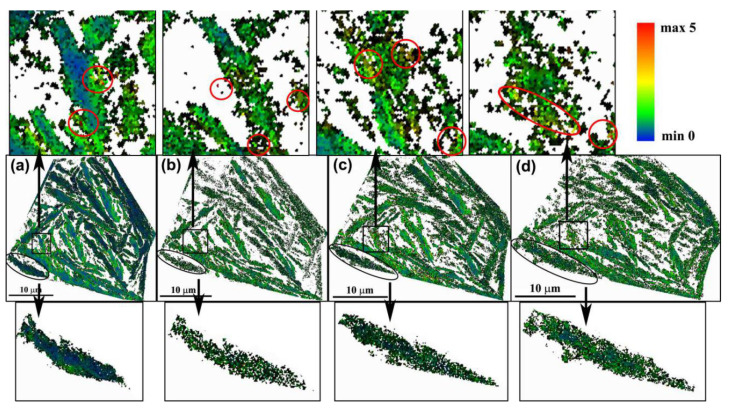
Distribution of KAM for bainitic ferrite (BF) at different strains: (**a**) 0, (**b**) 0.025, (**c**) 0.054, and (**d**) 0.104. Note: the red ellipses indicate the high-strain regions.

The KAM distributions for the RA and BF are shown in Figure 8 and Figure 9, respectively. The various KAM values are represented by a color gradient. In Figure 8a and Figure 9a, the KAM values at the phase boundaries are higher than the value interior of the plate. These findings indicate the existence of a strain field around the phase boundaries prior to the tensile process; and this strain field is introduced by the shear transformation and volume expansion of bainite phase transformation [30]. With increasing strain, the KAM values in the inner phase of RA and BF gradually increased. The KAM value was relatively uniform in RA, as shown in the rectangular area in Figure 8. By comparison, the KAM value in BF was asymmetrical, because of the presence several high-KAM-value regions. The high KAM value regions indicated a high microstrain at these regions. The number of high-strain regions increased with increasing macrostrain, as shown in the red ellipses in Figure 9. Previous studies revealed the presence of numerous C-clusters in BF, because of the low solubility of the C atom in body-centered cubic (bcc)-structured BF [31,32,33]. These C-clusters pin the dislocations, and hinder lippage, resulting in the strain concentration around the C-clusters, and the formation of the high-KAM-value regions.

**Figure 10:**
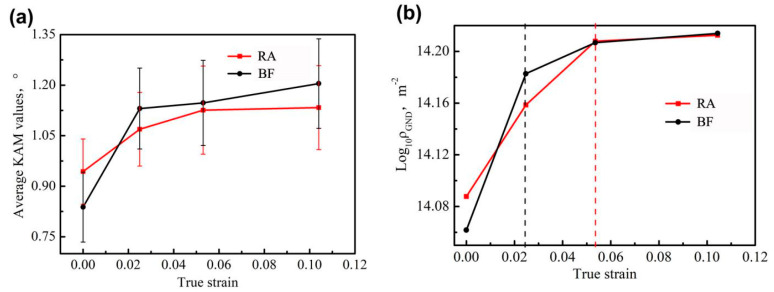
Variations of the KAM value (**a**) and logarithm of the geometrically necessary dislocations (log-GNDs) (**b**) in RA and BF, with true strains.

Figure 10 displays the variations in KAM value and GND density with increasing true strain. The KAM value of the BF quickly increased from 0.84° to 1.20° during early tensile process; the increase rate of the KAM value notably decreased. Correspondingly, the GND density in BF increased rapidly from 1.15 × 10^14^ to 1.62 × 10^14^, with increasing strain to 0.025, and then the increasing tendency of GND density slowed down. This pattern indicates that large deformation occurred in the BF at the early stage of tensile deformation, and increased dislocation density and hardness in BF. The mobility of dislocation in the bcc-structured BF was harder than that in the face-centered cubic (fcc)-structured RA. Unlike that of BF, the KAM value of RA indicated a constantly increasing rate within the true strain range of 0–0.054; beyond this range, the KAM value remained relative stable (Figure 10a). The variation tendency of GND density in the RA was similar to that of the KAM value, within 0–0.054. The increase rate of GND density markedly decreased when the true strain exceeded 0.054 (Figure 10b).

Fcc-structured RA contains more slip systems than bcc-structured BF, resulting in a strong deformability of RA. Moreover, RA possesses a stronger storage capacity for GNDs compared with BF. On the one hand, RA generates numerous GNDs because of its own deformation. On the other hand, RA absorbs the GNDs from the adjacent BF [19,21]. These two reasons result in a rapid increase of KAM value during 0–0.054. The notable hardening behavior of RA reduces further deformability. The absorption of dislocation by RA also decreases the increase rate of dislocation density in BF, indirectly reducing the increase rate of the KAM value.

Figure 5 shows the rapid decrease in RA at the early stage of deformation, owing to transformation into martensite. RA, which presents a high dislocation density, should be initially transformed into martensite during deformation. This effect indirectly decreases the increase rate of GND density in RA and is responsible for the lower increase rate of GND density in RA than in BF at the early stage of deformation.

The microstrain in each phase during deformation is inevitably heterogeneous, owing to different features of the phases. To analyze the variation tendency of the strain fraction for each phase, we calculated the KAM fraction of two phases through Equation (2):

(2) $$\begin{array}{l} f(RA)\%=\frac{KAM_{RA}}{KAM_{RA}+KAM_{BF}}\times 100\% \end{array}$$

where *KAM_RA_* is the KAM value of RA, and *KAM_BF_* is the KAM value of BF. The variation tendencies of the KAM fraction in the two phases are shown in Figure 10. Notably, the KAM fraction cannot represent the absolute strain fraction of materials, but its change reveals the development of strain fraction.

**Figure 11:**
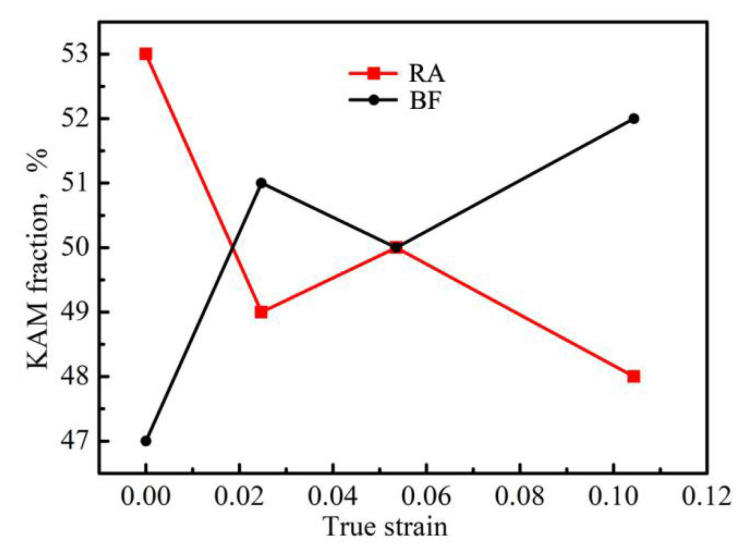
Variation in KAM fraction for each phase, with true strains.

Figure 11 shows the variation of KAM fraction for each phase with increasing true strain. The KAM fraction in RA was higher than that in the BF at the initial stage. With increasing true strain, the KAM fraction of the RA decreased, whereas the KAM fraction of the BF increased. The decreased KAM fraction of RA is due to two causes. First, the high dislocation density resulting from the deformation of itself, and the absorption from the neighboring BF, reduces the deformability of RA. Second, martensite transformation also strengthens RA, and therefore reduces the latter’s deformability. BF is the major phase in the microstructure, and inevitably absorbs additional strain from the specimen. Furthermore, the RA continually absorbs the dislocations of the adjacent BF and coordinates the deformation of the adjacent BF. These phenomena result in the increased KAM fraction of BF. The results also illustrate that the RA markedly contributes to the total deformation at the early deformation stage; however, this contribution weakens with increasing strain. BF, the major phase, increasingly contributes to intensifying strain under RA coordination.

## 4. Conclusions

In-situ EBSD was used to study the microscopic plastic deformation of RA and BF in medium carbon carbide-free bainitic steel. The martensitic transformation of RA and strain distribution between two phases were analyzed. The following conclusions can be drawn:

1. The transformation from RA into martensite occurs more quickly at the early stage (0–0.054) of deformation than at the later stage (0.054–0.104). The martensitic transformation of RA is notably affected by the orientation of the surrounding BF. The low SF value of the surrounding BF retarded martensitic transformation, whereas high SF value accelerated it.
2. The KAM value of RA was more symmetrical than that of the BF during tension. The true strain of 0.025 was the critical point for BF, because the KAM value and GND density increased rapidly before 0.025 and slowed down after 0.025. The variation tendency of KAM in RA was similar to that in BF, but the critical point was 0.054.
3. With increasing true strain, the KAM fraction of the RA decreased, whereas the KAM fraction of BF increased. This result reveals that the RA provided a higher contribution than BF at the early stage of tension to the whole deformation of the specimen, and that the contribution gradually decreased. As the major phase in the microstructure, BF contributed increasingly with intensifying strain under coordination with RA.

## Figures and Tables

**Table 1:** Main chemical composition of the test steel, wt.%.

C	Si	Mn	Cr	Ni	Mo	Al
0.49	1.60	1.62	1.28	0.34	0.39	0.57

## References

[1] Long X.Y., Zhang F.C., Kang J., Lv B., Shi X.B. (2014). Low-temperature bainite in low-carbon steel. Mater. Sci. Eng. A.

[2] Long X.Y., Kang J., Lv B., Zhang F.C. (2014). Carbide-free bainite in medium carbon steel. Mater. Des..

[3] Song C., Yu H., Li L. (2016). The stability of RA at different locations during straining of I & Q & P steel. Mater. Sci. Eng. A.

[4] Creuziger A., Foecke T. (2010). Transformation potential predictions for the stress-induced austenite to martensite transformation in steel. Acta Mater..

[5] Zhao J.L., Zhang F.C., Yu B.D., Liu H. (2017). Bainite and tempering stability in 70Si3MnCrMo steel. Iron Steel.

[6] Long X.Y., Zhang F.C., Kang J., Yang Z.N., Wu D.D., Wu K.M., Zhang G.H. (2016). Study on carbide-bearing and carbide-free bainitic steels and their wear resistance. Mater. Sci. Technol..

[7] Knijf D.D., Föjer C., Kestens L., Petrov R. (2015). Factors influencing the austenite stability during testing testing of Quenching and Partitioning steel by via in-situ Electron Backscatter Diffraction. Mater. Sci. Eng. A.

[8] Knijf D.D., Nguyen-Minh T., Petrov R.H., Kestens L.A.I., Jonas J.J. (2014). Orientation dependence of the martensite transformation in a quenched and partitioned steel subjected to uniaxial tension. J. Appl. Cryst..

[9] Nie W.J., Shang C.J., Guan H.L., Chen S.H. (2012). Structural regulation and deformation behavior of bainite ferrite/bainite (F/B) dual phase steel. Acta Met..

[10] Wei X., Fu L.M., Liu S.C., Wang W., Shan A.D. (2013). Deformation behavior of constituent phases and the affected factors in dual-phase steel. Chin. J. Mater. Res..

[11] Varshney A., Sangal S., Mondal K. (2016). Strain Partitioning and Load Transfer in Constituent Phases in Dual-Phase Steels. J. Mater. Eng. Perform..

[12] Wilkinson A.J., Britton T.B. (2012). Strains, planes, and EBSD in materials science. Mater. Today.

[13] Nye J. (1953). Some geometrical relations in dislocated crystals. Acta Met..

[14] Jiang J., Britton T., Wilkinson A. (2013). Measurement of geometrically necessary dislocation density with high resolution electron backscatter diffraction: Effects of detector binning and step size. Ultramicroscopy.

[15] Littlewood P., Britton T., Wilkinson A.J. (2011). Geometrically necessary dislocation density distributions in Ti–6Al–4V deformed in tension. Acta Mater..

[16] Field D., Trivedi P., Wright S., Kumar M. (2005). Analysis of local orientation gradients in deformed single crystals. Ultramicroscopy.

[17] Jiang J., Britton T.B., Wilkinson A.J. (2013). Evolution of dislocation density distributions in copper during Treadings. J. Acta Mater..

[18] Ramazani A., Mukherjee K., Schwedt A., Goravanchi P., Prahl U., Bleck W. (2013). Quantification of the effect of transformation-induced geometrically necessary dislocations on the flow-curve modelling of dual-phase steels. Int. J. Plast..

[19] Wang Y., Zhang K., Guo Z., Chen N., Rong Y. (2012). A new effect of retained austenite on ductility enhancement in high strength bainitic steel. Mater. Sci. Eng. A.

[20] Bingert J.R., Mason T.A., Kaschner G.C. (2002). Deformation twinning in polycrystalline Zr: Insights from electron backscattered diffraction characterization. Met. Mater. Trans. A.

[21] Zhang K., Zhang M., Guo Z. (2011). A new effect of RA on ductility enhancement in high-strength quenching-partitioning-tempering martensitic steel. Mater. Sci. Eng. A.

[22] Li W., Gao H., Nakashima H., Hata S., Tian W. (2016). In-situ study of the deformation-induced rotation and transformation of retained austenite in a low-carbon steel treated by the quenching and partitioning process. Mater. Sci. Eng. A.

[23] Wright S.I., Nowell M.M., Field D.P. (2011). A review of strain analysis using electron backscatter diffraction. Microsc. Microanal..

[24] Betanda Y.A., Helbert A.-L., Brisset F., Mathon M.-H., Waeckerlé T., Baudin T. (2014). Measurement of stored energy in Fe–48%Ni alloys strongly cold-rolled using three approaches: Neutron diffraction, Dillamore and KAM approaches. Mater. Sci. Eng. A.

[25] Zhilyaev A., Morozova A., Cabrera J.M., Kaibyshev R., Langdon T.G. (2016). Wear resistance and electroconductivity in a Cu–0.3Cr–0.5Zr alloy processed by ECAP. J. Mater. Sci..

[26] Li W.-S., Gao H.-Y., Nakashima H., Hata S., Tian W. (2016). In-situ EBSD study of deformation behavior of retained austenite in a low-carbon quenching and partitioning steel via uniaxial tensile tests. Mater. Charact..

[27] Takayama Y., Szpunar J.A., Kato H. (2005). Analysis of intragranular misorientation related to deformation in an Al-Mg-Mn alloy. Mater. Sci. Forum.

[28] Deng Y., Hajilou T., Wan D., Kheradmand N., Barnoush A. (2017). In-situ micro-cantilever bending test in environmental scanning electron microscope: Real time observation of hydrogen enhanced cracking. Scr. Mater..

[29] Petrov R., Kestens L., Zambrano-Robledo P., Guerrero M.P., Colás R., Houbaert Y. (2003). Microtexture of thin gauge hot rolled steel strip. Isij. Int..

[30] Xu G., Xu G., Mao X., Zhao G., Bao S. (2017). Method to evaluate the kinetics of bainite transformation in low-temperature nanobainitic steel using thermal dilatation curve analysis. Metals.

[31] Kang J., Zhang F., Long X., Lv B. (2016). Low cycle fatigue behavior in a medium-carbon carbide-free bainitic steel. Mater. Sci. Eng. A.

[32] Sourmail T., Smanio V. (2013). Low temperature kinetics of bainite formation in high carbon steels. Acta Mater..

[33] Timokhina I.B., Beladi H., Xiong X., Adachi Y., Hodgson P. (2011). Nanoscale microstructural characterization of a nanobainitic steel. Acta Mater..

